# PRECYCLE: multicenter, randomized phase IV intergroup trial to evaluate the impact of eHealth-based patient-reported outcome (PRO) assessment on quality of life in patients with hormone receptor positive, HER2 negative locally advanced or metastatic breast cancer treated with palbociclib and an aromatase inhibitor or palbociclib and fulvestrant

**DOI:** 10.1186/s13063-023-07306-z

**Published:** 2023-05-17

**Authors:** Tom Degenhardt, Peter A. Fasching, Diana Lüftner, Volkmar Müller, Christoph Thomssen, Christian Schem, Isabell Witzel, Thomas Decker, Hans Tesch, Sherko Kümmel, Christoph Uleer, Rachel Wuerstlein, Oliver Hoffmann, Mathias Warm, Norbert Marschner, Timo Schinköthe, Ronald E. Kates, Johannes Schumacher, Burkhard Otremba, Matthias Zaiss, Nadia Harbeck, Marcus Schmidt, on behalf of the PreCycle Investigators

**Affiliations:** 1 Breast Center, Department of Obstetrics and Gynecology and CCC Munich, LMU University Hospital, Munich, Germany; 2 Hausarztpraxis Wolfratshausen, Wolfratshausen, Germany; 3 Obstetrics and Gynecology, University Hospital Erlangen, Erlangen, Germany; 4 Immanuel Hospital Märkische Schweiz, Buckow, Germany; 5 Immanuel Hospital Rüdersdorf and Medical University of Brandenburg Theodor Fontane, Brandenburg, Germany; 6 Clinic and Polyclinic for Gynecology, Hamburg-Eppendorf University Medical Center, Hamburg, Germany; 7 Gynecology, Martin-Luther-University Halle-Wittenberg, Halle-Saale, Germany; 8 Mammazentrum, Jerusalem Hospital Hamburg, Hamburg, Germany; 9 Hematology/Oncology, Onkologie Ravensburg, Ravensburg, Germany; 10 Hämatologisch-Onkologische Gemeinschaftspraxis, Frankfurt, Germany; 11 Breast Center, Clinics Essen-Mitte, Essen, Germany; 12 Gemeinschaftspraxis Hildesheim, Hildesheim, Germany; 13 West German Study Group, Moenchengladbach, Germany; 14 Breast Center, University Hospital Essen, Essen, Germany; 15 Breast Center, Academic Hospital Cologne-Holweide, Cologne, Germany; 16 Study Coordination, iOMEDICO AG, Freiburg, Germany; 17 CANKADO Service GmbH, Kirchheim, Germany; 18 Research Center Smart Digital Health, University of the Bundeswehr, Neubiberg, Germany; 19 Statistics, Palleos Healthcare Gmbh, Wiesbaden, Germany; 20 Onkologische Praxis, Oldenburg, Germany; 21 Praxis Interdisziplinäre Onkologie U. Hämatologie, Freiburg, Germany; 22 Department of Obstetrics and Gynecology, University Medical Center of the Johannes Gutenberg-University, Mainz, Germany

**Keywords:** Metastatic breast cancer, eHealth, Patient-reported outcome, Quality of life, CDK 4/6 inhibitor, Endocrine therapy

## Abstract

**Background:**

Efficacy and quality of life (QoL) are key criteria for therapy selection in metastatic breast cancer (MBC). In hormone receptor positive (HR +) human epidermal growth factor receptor 2 negative (HER2 −) MBC, addition of targeted oral agents such as everolimus or a cycline-dependent kinase 4/6 (CDK 4/6) inhibitor (e.g., palbociclib, ribociclib, abemaciclib) to endocrine therapy substantially prolongs progression-free survival and in the case of a CDK 4/6i also overall survival. However, the prerequisite is adherence to therapy over the entire course of treatment. However, particularly with new oral drugs, adherence presents a challenge to disease management. In this context, factors influencing adherence include maintaining patients’ satisfaction and early detection/management of side effects. New strategies for continuous support of oncological patients are needed. An eHealth-based platform can help to support therapy management and physician–patient interaction.

**Methods:**

PreCycle is a multicenter, randomized, phase IV trial in HR + HER2 − MBC. All patients (*n* = 960) receive the CDK 4/6 inhibitor palbociclib either in first (62.5%) or later line (37.5%) together with endocrine therapy (AI, fulvestrant) according to national guidelines. PreCycle evaluates and compares the time to deterioration (TTD) of QoL in patients supported by eHealth systems with substantially different functionality: CANKADO active vs. inform. CANKADO active is the fully functional CANKADO-based eHealth treatment support system. CANKADO inform is a CANKADO-based eHealth service with a personal login, documentation of daily drug intake, but no further functions. To evaluate QoL, the FACT-B questionnaire is completed at every visit. As little is known about relationships between behavior (e.g., adherence), genetic background, and drug efficacy, the trial includes both patient-reported outcome and biomarker screening for discovery of forecast models for adherence, symptoms, QoL, progression free survival (PFS), and overall survival (OS).

**Discussion:**

The primary objective of PreCycle is to test the hypothesis of superiority for time to deterioration (TTD) in terms of DQoL = “Deterioration of quality of life” (FACT-G scale) in patients supported by an eHealth therapy management system (CANKADO active) versus in patients merely receiving eHealth-based information (CANKADO inform).

EudraCT Number: 2016–004191-22

## Background

Despite adjuvant therapy improvements in HR + /HER2 − breast cancer, a substantial proportion of patients still progresses to the metastatic stage. In the metastatic setting, when planning a therapy or therapy sequence, the focus is not only on efficacy, but also quality of life (QoL). Recently, some therapies have been approved for MBC to overcome hormone resistance such as everolimus or CDK 4/6 inhibitors (e.g., palbociclib, ribociclib, abemaciclib) which are administered orally.

Palbociclib is the first inhibitor of cyclin-dependent kinases (CDK) 4 and 6 that was approved in breast cancer. In vitro, palbociclib reduced cellular proliferation of ER-positive breast cancer cell lines by blocking progression of cells from G1 into S phase of the cell cycle. Based on the three large studies PALOMA-1, -2, and -3 [1–3], palbociclib was approved for pre- and postmenopausal patients with advanced/metastatic breast cancer who are candidates for aromatase inhibitor or fulvestrant.

The steady increase of oral drugs in anticancer treatment requires changes in patient management. Adherence to therapy over the entire course of treatment is a prerequisite for efficacy. While oral administration provides advantages compared to intravenous application of antineoplastic medications regarding QoL (e.g., flexibility, less wasted time and effort), patient responsibility is higher; there can be a loss of physician assistance and monitoring of the treatment. Maintaining adherence thus presents a challenge to disease management. In this context, factors influencing adherence include QoL factors such as patient satisfaction and early detection/management of side effects. New strategies for continuous support of oncological patients, particularly MBC patients, are needed. An eHealth-based platform such as CANKADO can help to support therapy management by probing the QoL status of the patient continuously throughout the course of treatment and, ideally, providing a basis for intensified care when indicated.

QoL combines different aspects of personal health status of an individual [4]. It represents a multi-domain concept, which includes the patient’s general perception of the effect of illness and treatment on physical, psychological, and social aspects of life. For cancer patients, it is important to determine further aspects such as economic burden, home management problems, or lack of emotional well-being—all of which can adversely affect QoL [5]. An important role of patient-reported outcome (PRO) measurement in cancer care is the determination of negative effects or the identification of needs for supportive care [6].

All these aspects are well covered in the FACT-G scale. The FACT-G as a part of the breast cancer questionnaire FACT-B is multidimensional, consisting of subscales assessing Physical Well-Being (PWB), Emotional Well-Being (EWB), Social Well-Being (SWB), and Functional Well-Being (FWB). The FACT-G yields a total score, as well as individual subscale scores, with higher scores reflecting better QoL [7, 8]. A change from baseline of 5 points or greater is considered a minimally important difference (MID) [9].

Having a continuous PRO feedback can heighten physicians’ awareness of their patients’ QoL [10]. A simplified PRO like a pain scale is feasible for daily documentation and is suited to improve communication between patients and healthcare professionals [11].

Additionally, uncertainty, lack of confidence, or anxieties related to the physician are factors that are known to influence outpatient communication [12]. These factors can even lead to a clinical phenomenon known colloquially as “white-coat hypertension,” particularly in older breast cancer patients (> 50 years) [13, 14]. More seriously, there are indications that patients may be inhibited to report clinical symptoms or discomfort in their entirety during examinations, due perhaps to a psychologically motivated desire to “please” their physician. eHealth platforms can serve to empower patients by overcoming uncertainty and anxiety in physician communication, lowering the threshold for describing symptoms, and ultimately providing more complete patient reports [6, 15].

The “ISPOR ePRO Good Research Practices Task Force” reported that data from an electronic PRO (ePRO) questionnaire adapted from a paper-based questionnaire had equivalent or superior performance (e.g., exhibited higher reliability) than the data from the original paper version [16]. Moreover, ePROs avoid data entry errors and reduce missing data as compared to paper-based PRO, provide immediate access to data, enable triggering alerts/notifications, and increase the patient’s willingness to report potentially sensitive information. In addition, data obtained from ePROs provide real-time tracking of survey compliance [6]. Remarkably, Basch et al. found that ePRO documentation is associated with improved overall survival compared to routine care in cancer patients [17].

Giving patients the opportunity to document complaints and QoL continuously at home provides a more detailed overview about their progress and can be used for directed questions from the physician. It also facilitates a more granular and reliable longitudinal overview. Such reports can improve the understanding of QoL of cancer patients receiving oral therapies.

The PreCycle trial was designed to evaluate the impact of ePROs in MBC using CANKADO (www.cankado.com). CANKADO is designed as an eHealth portal aimed to support therapy management, adherence, and physician–patient interaction [18]. Within PreCycle, CANKADO allows drug intake documentation, supports collection of ePRO data in a highly standardized manner, and provides overview reports to the investigators. These features have the additional advantage of promoting participant retention.

## Material/methods

### Study design

**Fig. 1:**
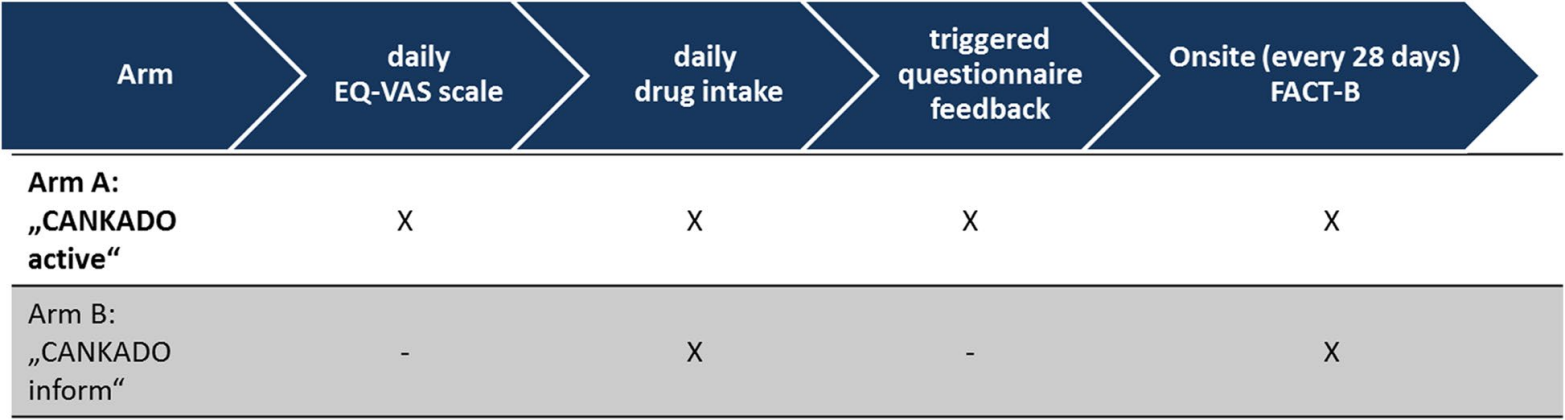
Schedule of ePRO documentation

**Fig. 2:**
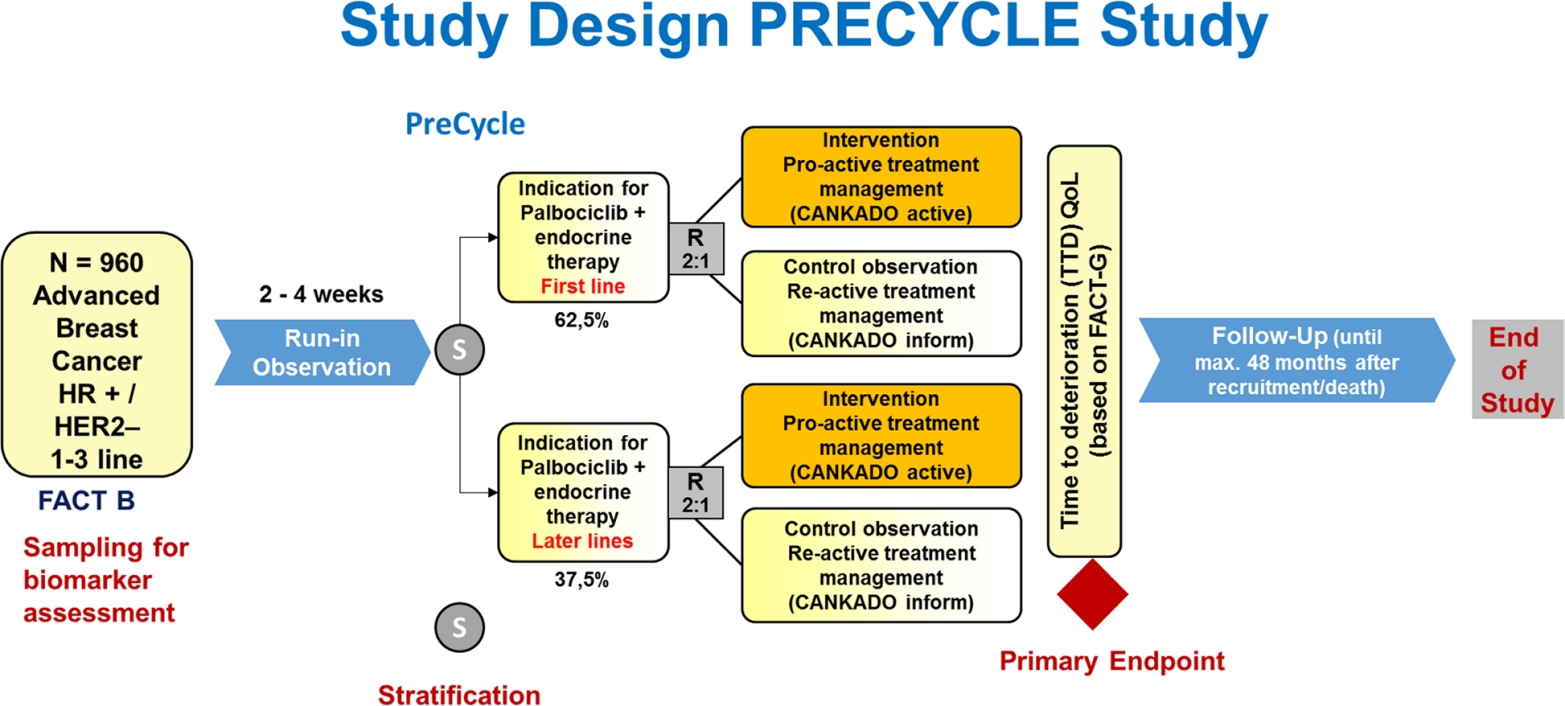
PreCycle—study design

PreCycle (Design: see Figs. 1 and 2) is a multicenter, randomized, parallel-group, phase IV clinical trial. The primary objective is to test the hypothesis of superiority regarding “time to deterioration” (TTD) of QoL for patients using the ePRO system “CANKADO active” compared to those using the “CANKADO inform” version. “CANKADO active” is the fully functional CANKADO-based eHealth treatment support service, including documentation of daily drug intake, daily documentation of QoL, feedback functions (PRO-React), and on-site surveys. “CANKADO inform” stands for a CANKADO-based eHealth service with a personal login. On-site surveys without feedback functions for the patient and documentation of daily drug intake are also available.

Information about data entry, coding, and security policies for the CANKADO eHealth system can be found at https://cankado.com/policy.

### Participants

Eligible patients have histologically or cytologically proven diagnosis of HR+ / HER2- locally advanced or metastatic breast cancer and are either candidates to receive palbociclib in combination with aromatase inhibitor or candidates to receive palbociclib in combination with fulvestrant for their locally advanced or metastatic disease. All anticancer treatments used in this study are approved drugs and therapy is in accordance to national treatment guidelines [19]. The trial compares two different kinds of eHealth support and documentation of patient-reported quality of life data.

There is no blinding process used in the study.

For inclusion and exclusion criteria, please refer to Table 1. In patients who are candidates for palbociclib in combination with aromatase inhibitor or fulvestrant, one prior line of chemotherapy for locally advanced or metastatic breast cancer is admissible, in addition to a maximum of two lines of endocrine therapy.

**Table 1:** PreCycle**—**inclusion and exclusion criteria

Inclusion criteria	Exclusion criteria
Post- or pre/peri-menopausal female patients, age ≥ 18 years	Known hypersensitivity to aromatase inhibitor, fulvestrant, palbociclib or any of its excipients
Patients with metastatic or locally advanced (non-operable) breast cancer disease	Contraindication for aromatase inhibitor, fulvestrant or palbociclib; or LHTH-agonists (if pre-menopausal)
Patients who are appropriate candidates for aromatase inhibitor + palbociclib combination therapy	Prior treatment with any CDK inhibitor
Patients having already received endocrine therapy who are appropriate candidates for fulvestrant + palbociclib combination therapy	Patients with locally advanced or metastatic, symptomatic, visceral spread, who are at risk of life threatening complications in the short term
One prior line of chemotherapy and/or a maximum of two endocrine therapy lines for locally advanced or metastatic disease is/are allowed	Known active uncontrolled or symptomatic CNS metastases
Peri-/pre-menopausal patients should additionally receive a LHRH-agonist	Current use of food or drugs known to be potent inhibitors or inducers of CYP3A4
The tumor must be hormone-receptor positive	High cardiovascular risk, including, but not limited to recent myocardial infarction, severe/unstable angina, or severe cardiac dysrhythmias in the past 6 months of enrollment
The tumor must be HER2-negative defined as either HER2 immunohistochemistry score 0 or 1 + or as HER2-negative by ISH	Diagnosis of any second malignancy within the last 5 years prior to enrollment, except for adequately treated basal cell or squamous cell skin cancer, or carcinoma in situ of the cervix
Eastern Cooperative Oncology Group (ECOG) performance status 0–2	Participation in other clinical trials involving investigational drug(s) (phases 1–4) within 2 weeks before the current study begins and/or during study participation
Tissue of the primary tumor and metastatic lesion for biomarker study if applicable	Lactating women
Adequate organ and marrow function	Life expectancy < 3 months
In case of patients of child bearing potential: negative serum pregnancy test at baseline. Patients must agree to use highly effective non-hormonal contraception	Known infection with HIV, hepatitis B virus, or hepatitis C virus
Resolution of all acute toxic effects of prior therapy, including radiotherapy grade < 1 (except toxicities not considered a safety risk for the patient) and recovery from surgical procedures	Concurrent severe, uncontrolled systemic disease, social or psychiatric condition that might interfere with the planned treatment and with the patient’s adherence to the protocol
Willingness and capability to use CANKADO	Legal incapacity or limited legal capacity
Availability of hardware: computer and/or tablet and/or smartphone with internet access	
Signed written informed consent	

Patients are stratified according to their eligibility for receiving palbociclib with endocrine therapy (AI or fulvestrant) as first or later lines.

The study includes a variety of different centers and clinical settings in Germany (university, community hospitals, private practice). More information including participating sites can be found at http://www.precycle.info.

### Treatment, stratification, randomization, data

Patients allocated to the combination of palbociclib with aromatase inhibitor receive:

- Palbociclib, 125 mg, orally once daily on day 1 to day 21 of every 28-day cycle followed by 7 days off treatment and
- Aromatase inhibitor, orally once-daily (continuously).
- Pre- or peri-menopausal patients should additionally receive a LHRH-agonist

Patients allocated to the combination of palbociclib with fulvestrant receive:

- Palbociclib, 125 mg, orally once daily on day 1 to day 21 of every 28-day cycle followed by 7 days off treatment and
- Fulvestrant, 500 mg, intramuscularly on days 1 and 14 of cycle 1, every 28 days (± 7 days) thereafter starting.
- Pre- or peri-menopausal patients should additionally receive a LHRH-agonist

Participants are randomly assigned to either experimental (“CANKDO active”) or control group (“CANKADO inform”) with a 2:1 allocation as per a computer-generated randomization schedule stratified by site and prior therapy line (first line vs. later line) using permuted blocks of random sizes. The block sizes are not disclosed, to ensure concealment. Randomization lists are created by the sponsor biostatistics department and forwarded to the sponsor randomization center. Figure 2 illustrates the expected patient distribution across strata and arms.

Patient assignment to the PreCycle study arms is managed centrally by a combined process involving the CANKADO system and the eCRF. After a patient has signed the ICF and screening measurements have been completed, patient baseline data have to be documented in eCRF by site personnel. Then, the site creates a patient account in CANKADO. The CANKADO system generates a patient-specific CANKADO trial ID which is part of the baseline documentation at the eCRF. The patient-specific CANKADO trial ID is the link between the two systems. When baseline documentation is completed, the site can request patient enrollment at the sponsor randomization center via phone or email. The randomization center assigns the study arm according to site specific randomization lists via eCRF and in parallel to the patient account in the CANKADO system. Afterwards, the site activates patient’s CANKADO account.

Patients continue to receive study treatment together with the assigned ePRO assessment until investigator assessed disease progression, symptomatic deterioration, unacceptable toxicity, death, or withdrawal of consent, whichever occurs first.

Adverse events (AEs) and serious adverse events (SAEs) are collected throughout palbociclib treatment and 28 days after the last dose. When an AE/SAE occurs, it is the responsibility of the investigator to review all documentation (e.g., hospital progress notes, laboratory reports, and diagnostics reports) related to the event. The investigator then record all relevant AE/SAE information in the eCRF.

Patients discontinuing the active treatment phase enter a follow-up period phase; further progression and new anti-cancer therapy information are collected once a year up to 48 months after randomization.

**Fig. 3:**
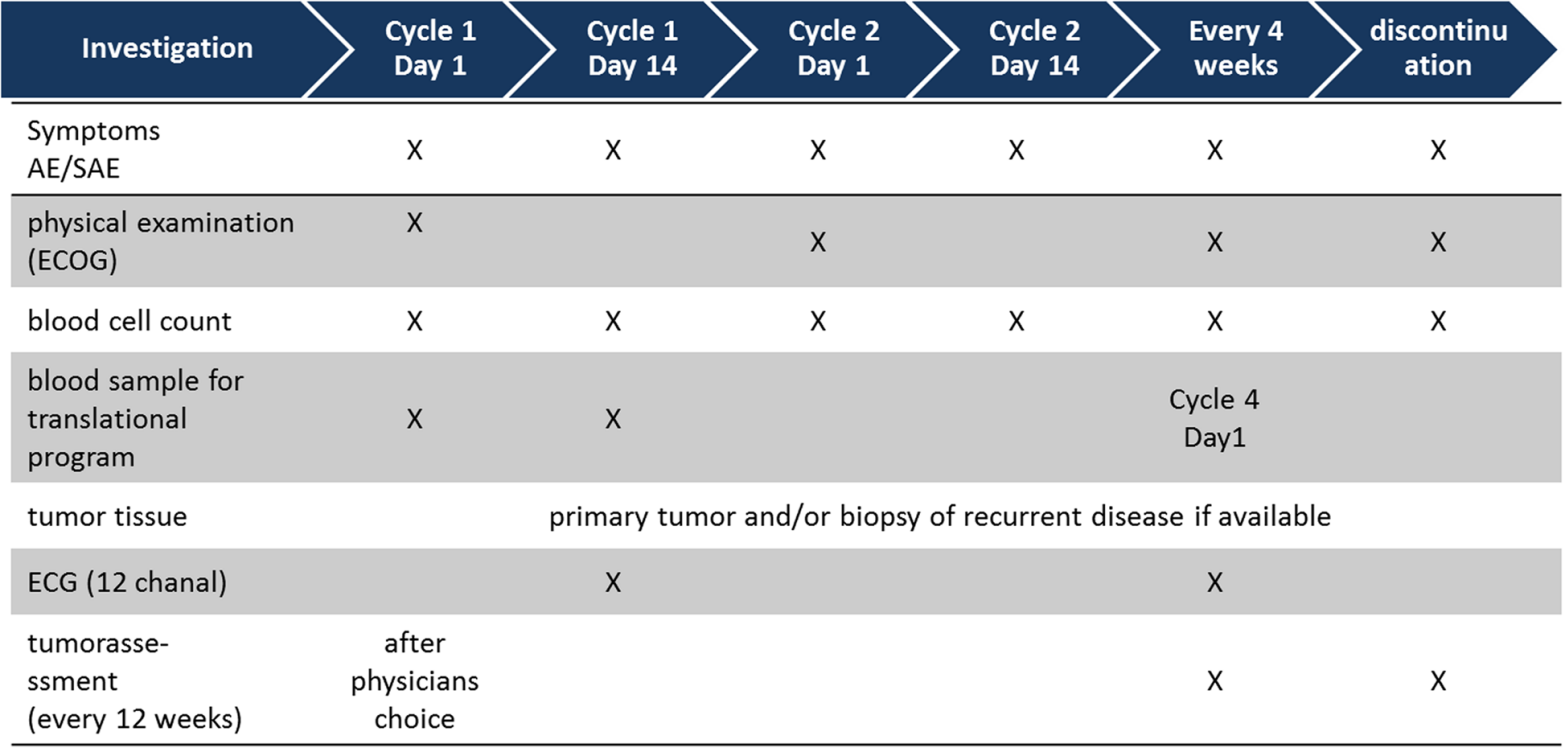
PreCycle—schedule of investigations

In addition, biomarkers are assessed as a scientific translational program within this study. Tumor material from available primary tumor and/or available biopsies from recurrent disease are collected. Blood samples are collected at four time points during the study when also routine blood samples are mandatory (see Fig. 3).

All study treatments are approved treatments following clinical standards and local guidelines.

### Statistical considerations

The primary endpoint is time to deterioration (TTD) of quality of life (QoL), based on the FACT-G instrument total score. This time is defined as the interval from registration until a DQoL (deterioration of quality of life) event, determined using the FACT-G scale (or censoring). Measurements are taken at day 1 of each 28-day treatment cycle in both arms. The event “deterioration of quality of life (DQoL)” is defined as any decrease of 10 or more points from baseline unless a recovery is achieved in the subsequent assessment. A recovery is defined as a QoL score no worse than 9 points below baseline. If data of the subsequent visit is missing, a decrease of 10 or more points will be considered as an event. The study tests the hypothesis of superiority of TTD in patients supported by eHealth therapy management in arm A compared to arm B.

Sample size was estimated as follows: the superiority hypothesis test for DQoL is based on the stratified score function test of a Cox regression model with Breslow likelihood, including therapy line stratum as covariate. Assuming a (constant) hazard ratio of 0.8 for TTD in arm A vs. arm B, the study is designed to refute the null hypothesis of equal survivor functions between arm A and arm B at a two-sided 5% significance level [20, 21] with at least 80% power.

Patients in the strata defined above are referred to here as “first-line” and “later-line” patients. First-line patients are assumed to comprise about 5/8 (62.5%) of the entire trial population; the remaining patients (3/8, i.e., 37.5%) are assumed to be treated in later lines. In first-line patients, the proposed hazard ratio between CANKADO arms of 0.8 corresponds to about 4 months superior TTD for CANKADO active; in later-line patients, it corresponds to about 2 months superior TTD. Such an increase is assumed to have a clinically relevant benefit.

To estimate a *lower* bound for the expected number of DQoL (deterioration) events, we assume that, at the very least, disease progression will generate a DQoL event. Therefore, the median PFS reported in the PALOMA-1 and -2 trials (first-line patients treated with palbociclib and letrozole) [1, 2], as well as PALOMA-3 (2nd line patients treated with palbociclib and fulvestrant) [3] may serve as model for first-line and later-line strata here. Consequently, we used the upper confidence limit for median PFS reported in PALOMA-1 (27.5 months) [1] and PALOMA-3 (11 months) [3] to compute conservative estimates for the expected number of events in the first-line and later-line stratum respectively.

During the conduct of the study no interim analysis is planned.

The primary analysis implements the intent-to-treat principle (ITT). Primary reporting is based on the ITT population of all randomized patients, including patients with protocol violations and intercurrent events, handled by a while-on-treatment strategy. An additional sensitivity analysis will be carried out based on a per-protocol population excluding patients with major protocol violations (to be determined during data review prior to database lock based on prospectively defined criteria). For the primary analysis, missing values are treated as missing-at-random by therapy line and treatment arm strata. Patient information entering the analysis will be either time of censoring (e.g., administrative censoring or intercurrent event) or the actual event time.

The sample size was estimated using a validated Monte-Carlo simulation implemented in Python 3.5. In all 4 groups (2 arms with 2 strata each), exponential survival was used as parametric sampling distribution with hazard rate computed from median PFS estimates as indicated above. In addition, an independent exponential censoring process was used to simulate loss to follow-up with 48-month probability of censoring calibrated at 10%.

If 960 patients are recruited (assuming 10% loss to follow-up), we can expect to reject the null-hypothesis with 80% power if a stratified two-sided test of equal hazards between (CANKADO active) and (CANKADO inform) is performed at alpha = 0.05. The corresponding expected number of events across groups is 693.

For each stratum and arm, Kaplan–Meier product-limit estimators of the survivor functions together with Hall-Wellner confidence bands will be reported and visualized graphically. In addition, corresponding median TTD with log-transformed 95% confidence will be reported.

Secondary endpoints include a definition of TTD of QoL as minimally important difference (5-point drop from baseline on FACT-G scale) to provide a sensitivity analysis with respect to the primary endpoint, progression-free survival and overall survival as clinical outcomes, as well as ePRO measurements of global health status and FACT-B scales. Translational research questions will focus on the influence of genetic and immune biomarker profiles on clinical outcome.

## Discussion

Since MBC is a chronic disease, maintaining a good QoL is of foremost importance. Enrolled patients receive an approved therapy (aromatase inhibitor + palbociclib or fulvestrant + palbociclib) in both arms of this randomized study. Potential risks (e.g., toxicity) should be equally distributed between both arms. A theoretical, albeit unlikely, risk might be that an eHealth-based high density observation using CANKADO could have a negative impact on clinical outcome or QoL. Therefore, the primary objective is to demonstrate superiority of time to deterioration (TTD) of quality of life for patients with eHealth-based high-density observation using CANKADO (CANKADO active) versus eHealth-based static observation on site (CANKADO inform). This clear focus on QoL should provide a benefit for all patients enrolled to this trial.

To our knowledge, PreCycle is the largest world-wide trial evaluation of the benefits of an eHealth therapy support in oncology. This trial should lead to an increased awareness of eHealth tools like CANKADO to monitor QoL under systemic treatment. Continuous PRO documentation may lead to increased patient empowerment in oncology and addresses an urgent need as oral therapies are becoming much more frequent [22]. ePROs have the potential improving patient-physician communication while individualizing site visits without compromising patient safety. PreCycle will address the impact on patient QoL of such continued ePRO documentation and thus add to the knowledge-base in the literature. The accompanying translational research program is implemented into the study design to improve our understanding of the mechanisms of resistance to endocrine therapies.

PreCycle started recruitment in mid-2017 and has already recruited almost 500 patients.

## Trial status

PreCycle: Multicenter, randomized phase IV intergroup trial to evaluate the impact of eHealth-based patient-reported outcome (PRO) assessment on quality of life in patients with hormone receptor positive, HER2 negative locally advanced or metastatic breast cancer treated with Palbociclib and an aromatase inhibitor- or Palbociclib and Fulvestrant.

Protocol Number: PH001PreCycle, Version 2.0

AGOB002 TraFO002-16

EudraCT Number: 2016–004191-22

Testing Objective: eHealth-based Patient-Reported Outcome (ePRO)

Study Treatment: Palbociclib in combination with endocrine therapy (aromatase inhibitor or / fulvestrant combined with a LHRH agonist in pre- or peri-menopausal women)

Sponsor Name and Legal Registered Address:

palleos healthcare GmbH

Taunusstr. 5a

65183 Wiesbaden

Short Title:

Impact of eHealth-support on Quality of Life in metastatic breast cancer patients treated with Palbociclib and endocrine therapy.

**Table:** 

Regulatory	Date
Approval of legal authority: Bundesamt für Arzneimittel und Medizinprodukte	05/19/2017
Approval ethics committee	07/06/2017
1^st^ data and safety monitoring board meeting	06/26/2017
1^st^ patient in	08/07/2017
App. end of recruitement	QII/2023
Study duration	48 months of follow-up

## Data Availability

The sponsor is committed to following high ethical standards for reporting study results for its innovative medicine, including the timely communication and publication of clinical trial results, whatever their outcome.

## Abbreviations

CDK: Cyclin-dependent kinase
DQoL: Deterioration of quality of life
HR +: Hormone receptor positive
MBC: Metastatic breast cancer
MID: Minimally important difference
OS: Overall survival
PRO: Patient-reported outcome
PFS: Progression-free survival
TTD: Time to deterioration
QoL: Quality of life
